# Consumer empowerment and self-assessment of empowerment

**DOI:** 10.1371/journal.pone.0259971

**Published:** 2021-11-12

**Authors:** Su-Jung Nam

**Affiliations:** Department of Home Economics Education, College of Education, Jeonju University, Jeonju, South Korea; St John’s University, UNITED KINGDOM

## Abstract

This study examined the influence of consumer empowerment and its self-assessment on consumers’ information search behavior and consumer life satisfaction; it also examined whether the results were consistent with the Dunning−Kruger effect. A total of 977 consumers who participated in a national consumer survey were divided into four groups, based on their level of empowerment and self-assessment. The Dunning−Kruger effect was observed in the consumer empowerment results, with 35.9% of respondents showing imbalanced empowerment and self-assessment levels. A general linear model was used to examine the survey results, which indicated that the main effect of empowerment had no significant effect on information searching or consumer life satisfaction. However, there was a significant main effect of self-assessment on both dependent variables. In addition, the interaction of empowerment and self-assessment had a significant effect only on information search behavior. Consequently, it can be concluded that self-assessed empowerment, rather than actual consumer empowerment, affects information search and consumer life satisfaction.

## Introduction

Consumer empowerment is a multifaceted concept encompassing a consumer’s skills, competencies, and rights. It also includes the consumer’s ability to gather and use information and the capacity of the market to provide legal and practical protection devices [1]. The interest in and debate regarding consumer empowerment has rapidly increased over the last two decades. Research has shown an association between consumer empowerment and consumers’ skills, competences, rights, and abilities, as well as greater consumer choice [2]. Additionally, consumer empowerment can be expanded to a concept that includes consumers’ active participation in production processes and reflects what consumers really want in products and services [3].

Consumer empowerment has been used in marketing literature [4] to indicate both a subjective state or experience related to an increase in consumer abilities [5] and an objective condition related to the consumer’s greater knowledge or understanding [6, 7]. In the latter concept, it has been found that a wider range of choices, easier access to information, and higher levels of consumer education are the prerequisites to empowerment, resulting in greater consumer involvement [8]. Consumer empowerment has further resulted in boycotts and protests, punishing producers deemed unethical and rewarding suppliers who demonstrate genuine ethical credentials [9].

Previous studies have revealed that consumer empowerment positively affects a consumer’s search for information [10, 11] and consumption satisfaction [5, 12–14]. However, although consumer empowerment offers numerous potential benefits, it does not always guarantee consumers’ successful (i.e., efficient and rational) decision making and effective information searches [15].

Consumers’ efficient and rational decision making is likely to be affected by the Dunning–Kruger effect [16], according to which, there is a cognitive bias in which people with low ability at a task overestimate this ability. The effect is related to the cognitive bias of illusory superiority and comes from people’s inability to recognize their lack of competence. Without an accurate self-assessment of metacognition, people cannot objectively evaluate their competency. Therefore, if consumer empowerment is understood and interpreted without considering a consumer’s self-assessment of their metacognition, there is a possibility of reaching erroneous or distorted conclusions. To accurately understand consumer empowerment, self-assessment of metacognition must be considered.

### Consumer empowerment

There is no academic consensus on or accepted definition of the concept of consumer empowerment. Instead, consumer empowerment is a term that frequently includes the related concepts of consumer ability, capacity, and competency. Regarding consumer education, consumer competency is a concept used to explain the overall competence that a consumer should have [1]. However, consumer ability or consumer capacity are mainly used as concepts referring to a consumer’s cognition, information searching, and information understanding abilities [17]. Based on previous studies [1, 6, 18, 19], consumer empowerment can be described as a consumer having the complete ability to achieve personally astute and socially sustainable consumption. In other words, consumers possess the knowledge, purpose, motivation, and ability to strive for their personal benefit as well as to influence producers [8].

In addition, consumer empowerment is a term used in the European Union’s (EU’s) Consumer Policy Strategy [1]. This term encompasses not only the ability of consumers to use information but also the technology and consumer participation to protect consumers’ rights and markets. According to the EU Consumer Policy Strategy 2007–2013 [1], empowered consumers need choices, accurate information, market transparency, and the confidence that comes from effective protection of consumer rights. Consumer empowerment tends to increase consumers’ knowledge, skills, and assertiveness [1] and can originate from different sources, including consumer education, valuable information, and institutional regulations. In particular, the EU Consumer Policy Strategy stated that the following elements are necessary when defining empowerment: consumers should be able to 1) make informed decisions when buying (e.g., reading terms and conditions, comparing prices, and inspecting product labels); 2) acquire information on their rights; and 3) have access to necessary advocacy and redress mechanisms [1]. In 2015, the Ministry of Trade Republic of Indonesia formed the Committee of Consumer Empowerment Index to measure consumer empowerment as an indicator of consumer welfare. The Indonesian Consumer Empowerment Index includes measures of a consumer’s awareness, understanding, and abilities at the stages before, during, and after the purchase [19].

In 2010, the Korea Consumer Agency (KCA) conducted national surveys, including a Delphi survey of experts and a survey of general consumers. The results of these surveys were updated in 2014 and 2018 and were used in 2018 to develop a Consumer Empowerment Index that measures consumer knowledge, attitudes, and behavior [20].

In general, consumer empowerment is based on three perspectives: knowledge, attitude, and function [21]. Knowledge can be easily measured and changed through learning. Conversely, attitude is difficult to measure and cannot be easily developed or changed in a short period of time; however, it influences the promotion of successful behavior. Therefore, improving consumers’ welfare is difficult if their knowledge, attitude, or behavior is overlooked when investigating consumer empowerment [17].

### Dunning−Kruger effect

The Dunning−Kruger effect is a type of cognitive bias that describes people’s belief that they are smarter and more capable than they essentially are. People with low capabilities do not possess the ability to recognize their own incompetence. The combination of poor self-awareness and low cognitive ability leads them to overestimate their capabilities. Kruger and David [16] explained that this bias results from an internal illusion in people with lower abilities and an external misperception in people with higher abilities, and that the causes of such cognitive bias are rooted in metacognition. To accurately assess one’s abilities related to a task, it is necessary to possess expertise in that task. However, people with lower abilities do not have this expertise, and therefore, cannot accurately assess their abilities. Dunning et al. [22] referred to this problem as a double curse. Conversely, people with higher abilities overestimate others’ abilities and erroneously assume others to have the same level of ability as themselves. As a result, the miscalibration of incompetent people stems from an error in self-perception, whereas the miscalibration of highly competent people stems from an error in their perception of others [16].

### Conceptual framework

**Fig 1:**
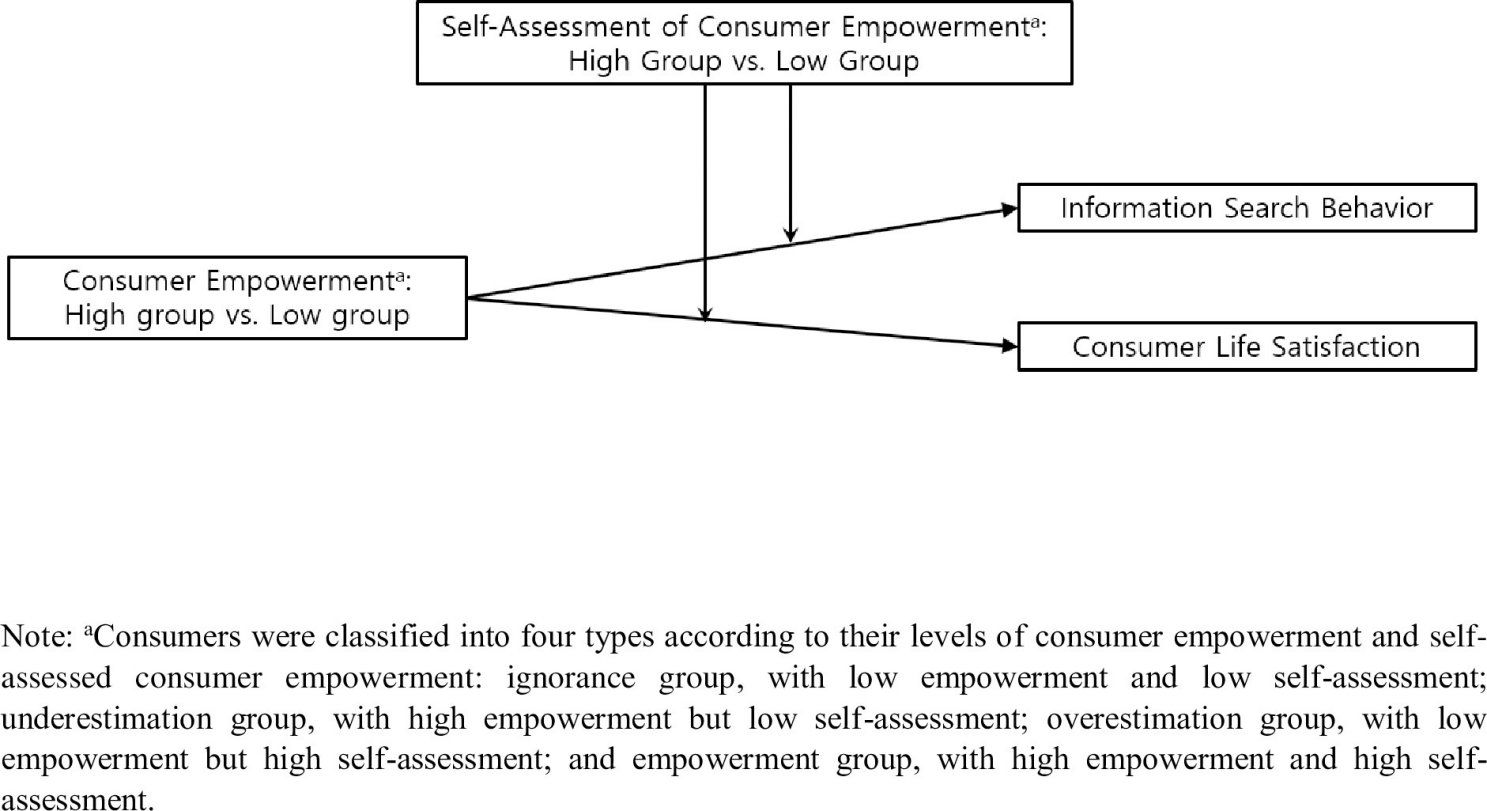
Conceptual diagram.

This study examined how the Dunning−Kruger effect applies to consumer empowerment. To this end, the study explores the moderating effect of self-assessment of consumer empowerment on the relationship between consumer empowerment, information search, and consumer life satisfaction, as shown in Fig 1. To clarify the above, this study poses the following research questions: (a) How are consumers classified for data analysis purposes? (b) What are the factors that influence the classification of consumer groups based on consumer empowerment and its self-assessment? (c) What are the main effects of consumer empowerment and its self-assessment on information searching and consumer life satisfaction? (d) What are the interactive effects of consumer empowerment and its self-assessment on information searching and consumer life satisfaction?

#### The relationship between consumer empowerment and information searching

Consumers’ information search behavior depends on their ability to detect and identify information [23, 24]. In particular, within consumer empowerment, consumer knowledge is highly related to information searching [23–25] and has been shown to influence information searching [26–28]. It has also been proven that, compared to other variables such as attitude and behavior, consumer knowledge has a far greater influence on information searching [29]. A study on adolescents’ clothing decision making by Joo et al. [30] showed that consumer empowerment had a positive correlation with information searching. Further, in Hwang and Kim’s research on consumer empowerment and its impact on the use of electronic products, it was found that consumer empowerment affects information search efficiency and effectiveness [31]. Thus, consumer empowerment can be expected to have a significant influence on information search behavior. Therefore, Hypothesis 1 was established based on the preceding studies.

***Hypothesis 1*.** Consumer empowerment has a positive effect on information search behavior.

#### The relationship between consumer empowerment and consumer life satisfaction

The concept of life satisfaction first appeared in Neugarten, Havinghurst, and Tobin, who used it as a dependent variable to verify the activity and separation theories, in 1961 [32]. They defined life satisfaction as a subjective assessment of one’s overall life, indicating successful adaption to the environment during the course of one’s life. Consumers’ life satisfaction is a subjective assessment of consumption experiences or situations whereby consumers manage their daily lives [33]. In Statistics Korea’s annual report of a social survey, consumer life satisfaction was defined as “satisfaction with daily life as a consumer,” indicating that it refers to satisfaction in “consumer life as a consumer” [34].

Consumer empowerment results in the ability to lead a reasonable life as a consumer; therefore, consumers who are highly empowered show a higher degree of rational consumer decision making, which is likely to result in higher consumption satisfaction [35]. Yoo et al. reported that consumer empowerment has a direct positive effect on overall consumer life satisfaction [36]. In previous studies, it was reported that financial empowerment affects satisfaction with economic life [37–39], and consumer citizenship empowerment has a positive influence on ethical product satisfaction [40]. Similarly, Lee and Lee used a path model to demonstrate how consumer empowerment indirectly affected satisfaction with medical services by acknowledging consumer rights in medical services [41]. Kim and Cho also examined users’ satisfaction with telecommunication services and reported that a user’s knowledge of telecommunication services, that is, the cognitive aspect of consumer empowerment, had a constant impact on their satisfaction with information provision and quality, proving that higher levels of knowledge lead to higher levels of satisfaction [42]. Based on the above, consumer empowerment can be expected to influence consumer life satisfaction. Therefore, Hypothesis 2 was established as follows.

***Hypothesis 2*.** Consumer empowerment has a positive effect on consumer life satisfaction.

#### The effect of empowerment self-assessment

Several studies conducted on a North American population have confirmed the Dunning−Kruger effect; however, studies from other cultures have produced mixed results [43]. Hein et al. reported that Japanese people tend to underestimate their abilities and view underachievement or failure as an opportunity to improve their abilities at a given task, thereby increasing their value to their social group [44]. In a study on Korean university students, Park verified the Dunning−Kruger effect and emphasized the significant influence of metacognition [45]. Moreover, research on political ignorance in Korea by Kim and Lee verified the Dunning−Kruger effect in political communication [46]. Individuals with moderately low political expertise rate themselves as increasingly politically knowledgeable when partisan identities are made salient. This below-average group is also likely to rely on partisan source cues to evaluate the political knowledge of peers [47]. A study by Hwang and Nam further distinguished between objective and subjective knowledge and identified how an imbalance between the two influenced consumers’ attitudes and purchase intentions toward genetically modified foods [48]. Various empirical studies have shown that individuals with low levels of competence will judge themselves to be more competent than they really are, whereas those with high levels of competence will underestimate their abilities; these studies emphasized the importance of self-assessment of consumer empowerment. Based on the above, the relationship between consumer empowerment, consumer information search behavior, and consumer life satisfaction can be assumed to differ, depending on the self-assessment of consumer empowerment. Therefore, Hypotheses 3 and 4 are proposed as follows.

***Hypothesis 3*.** The relationship between consumer empowerment and information searching depends on the self-assessment of consumer empowerment.

***Hypothesis 4*.** The relationship between consumer empowerment and consumer life satisfaction depends on the self-assessment consumer empowerment.

## Methods

### Data

This study used data from the *2018 Survey on Consumer Empowerment Index* conducted by the Korea Consumer Agency (KCA). From July 9 to August 8, 2018, 2,000 Korean respondents over the age of 20 completed a self-administered structured survey through face-to-face interviews with interviewers trained in consumer empowerment. The researcher then employed a secondary data analysis of the survey results and conformed to ethical standards. The results were analyzed for 977 participants; responses from 1,023 participants were excluded, who had reported “medium” self-assessment of consumer empowerment. Table 1 presents the participants’ characteristics.

**Table 1:** Participants’ characteristics (n = 977).

Characteristics	*n* (%)	
**Gender**		
Male	490 (50.2)	
Female	487 (49.8)	
**Age (years)**		
20−29	177 (18.1)	
30−39	173 (17.7)	
40−49	206 (21.1)	
50−59	187 (19.1)	
60 and above	234 (24.0)	
**Education**		
High School	424 (43.4)	
College	536 (54.9)	
Graduate	17 (1.7)	
**Monthly income** ^**a**^		
≤150	56 (5.7)	
150−300	218 (22.3)	
301−450	329 (33.7)	
451−600	244 (25.0)	
601−750	102 (10.4)	
751≤	28 (2.9)	
	**Range**	**M (SD)**
**Information search behavior**	1−10	6.34 (1.50)
**Consumer life satisfaction**	1−10	6.17 (1.47)

Note

^a^The unit is South Korean 10,000 won (KRW 10,000 = USD 8.90).

### Measures

The KCA conducted an expert Delphi survey based on the consumer empowerment measurement suggested by Lee and developed a scale for consumer empowerment; they published the *2010 Survey on Consumer Empowerment Index*, to be used for the first time in 2010 [45]. Since the development of the first Korean consumer empowerment scale in 2010, the validity and reliability of consumer empowerment measurements have been enhanced by conducting general consumer surveys and a Delphi survey of experts in 2014 and 2018. In this study, the third edition of the *2018 Survey on Consumer Empowerment Index* was used. It was developed on the basis of previous studies [1, 6, 18, 19] while maintaining consistency with the 2010 and 2014 consumer empowerment surveys and improving the content validity and reliability thereof.

Empowerment was measured by assessing consumers’ knowledge, attitudes, and behavior. For knowledge, respondents answered eight true-or-false questions on objective facts. The participants received 1 point for a correct answer and 0 for an incorrect one (see S1 Table). The mean scores of attitudes and behavior were calculated by measuring the respondents’ average levels of agreement with nine statements rated on a 5-point Likert scale, with Cronbach’s *α* = .788 and *α* = .813 for the attitude and behavior items, respectively (see S2 Table). Respondents were classified into the “high” group if their score was higher than the median value (i.e., 11.33) for the sum of knowledge (M = 4.03, SD = 1.43, min = 0, max = 8), attitude (M = 3.87, SD = .52, min = 1, max = 5), and behavior (M = 3.58, SD = .61, min = 1, max = 5) and into the “low” group if their score was lower than the median value.

Self-assessment was measured using a single self-report item with a five-point Likert scale: “What do you think of your overall level of consumer empowerment?” Of the 2,000 respondents, 1,023 who answered “medium” were excluded from the analysis, and data for the remaining 977 respondents were analyzed. Those who answered “very low” and “low” were classified into the “low” group, and those who answered “very high” and “high” were classified into the “high” group.

Information search behavior was calculated by measuring the respondents’ level of agreement with three statements (i.e., I tend to look for consumer information in newspapers, television, and radio with interest; I tend to look for consumer-related information on the Internet; and I tend to look for consumer information provided by government agencies), using a 10-point Likert scale, with Cronbach’s *α* = .772.

Previous studies have shown that a single-item self-report measure of life satisfaction is sufficient in terms of validity and reliability in certain research contexts [49–51]. In addition, many previous studies [34–42, 52], including the social survey satisfaction of the Korean National Statistical Office, have measured consumer life satisfaction as a single item. Therefore, in this study, consumer life satisfaction was assessed based on answers to a single item using a 10-point Likert scale: “How would you rate your satisfaction with daily life as a consumers?”

### Analysis

This study divided consumers into four groups according to their levels of empowerment and self-assessment: ignorance group (IG), with low empowerment and low self-assessment; underestimation group (UG), with high empowerment but low self-assessment; overestimation group (OG), with low empowerment but high self-assessment; and empowerment group (EG), with high empowerment and high self-assessment. To reveal the causal relationship between each factor and the four groups, a multinominal regression analysis was performed using gender, age, education, and monthly income as independent variables and consumer group as the dependent variable to reveal the factors influencing the classification of the consumer groups. Further, a general linear model was used to examine the effects of empowerment and self-assessment on consumers’ information search behavior and consumer life satisfaction. The analyses were conducted using SPSS Statistics 24.0 software.

## Results

### Consumer groups according to the level of empowerment and its self-assessment

Table 2 shows the distribution of consumers into the four groups according to their levels of empowerment and self-assessment of empowerment. Overall, 136 consumers (14.3%) were classified into IG, 63 (6.6%) into UG, 279 (29.3%) into OG, and 475 (49.8%) into EG. The χ^2^ value for the group division was 62.901, which was statistically significant (p < .001). Hence, EG was found to have the highest proportion of respondents, whereas UG had the lowest. The results indicated that 20.9% (i.e., IG = 14.3%, UG = 6.6%) of the respondents had low empowerment, and 35.9% (i.e., UG = 6.6%, OG = 29.3%) had imbalanced empowerment and self-assessment levels.

**Table 2:** Distribution of participants among consumer groups.

		Empowerment		
		Low	High	
		n (%)	n (%) χ^2^	
Self-assessment	Low	Ignorance Group (IG)	Underestimation Group (UG)	62.901***
		136 (14.3%)	63 (6.6%)	
	High	Overestimation Group (OG)	Empowerment Group (EG)	
		279 (29.3%)	475 (49.8%)	

Note

***p < .001.

### Determinants of consumer groups

Table 3 shows the multinomial regression results for the factors that influenced the classification of consumer groups. Specifically, the independent variables included in the multinomial regression model were gender, age, education, and monthly income.

**Table 3:** Multinomial regression of the consumer group.

	Underestimation group				Overestimation group				Empowerment group			
	(n = 63)				(n = 279)				(n = 475)			
	*B*	SE	Exp(B)	p	*B*	SE	Exp(B)	p	*B*	SE	Exp(B)	p
Male	.243	.311	.976	.096	.162	1.177	.1.177	.469	.088	.220	.1.092	.689
Age	-.024	.014	1.275	.435	-.055	.010	.946	.000	-.077	.010	.926	.000
College and above	-.016	.415	.984	.969	.102	.287	1.107	.723	.422	.278	1.526	.128
Mid income^a^	-.015	.339	.985	.964	.484	.253	1.623	.055	.649	.249	1.913	.009
High income^b^	-20.637	1.090E-9	.000	-^c^	.785	.415	2.192	.058	.939	.406	2.558	.021

*Notes*: Reference group = ignorance group (n = 136); -2Log likelihood = 1.303E3; Chi-square = 219.659 (*df* = 15, p < .000)

^a^KRW 301–600

^b^KRW 601 and above

^c^All participants in the underestimation group had high income, and therefore, p statistics for high income in the underestimation group were not calculated.

There were no significant effects of gender and education on consumer group classification. Age negatively affected the probability of being classified into OG and EG. Younger consumers were more likely to be classified into OG (B = -.055, Exp(B) = .946, p < .001) and EG (B = -.077, Exp(B) = .926, p < .001) than into IG. Mid-monthly income showed a more positive effect on the probability of consumers being included in EG (B = .649, Exp(B) = 1.913, p = .009), as compared to IG. Similarly, high monthly income showed a more positive effect on the probability of classifying consumers into EG (B = .939, Exp(B) = 2.558, p = .021) as compared to IG.

**Effects of empowerment and self-assessment on information search behavior and consumer life satisfaction**

**Fig 2:**
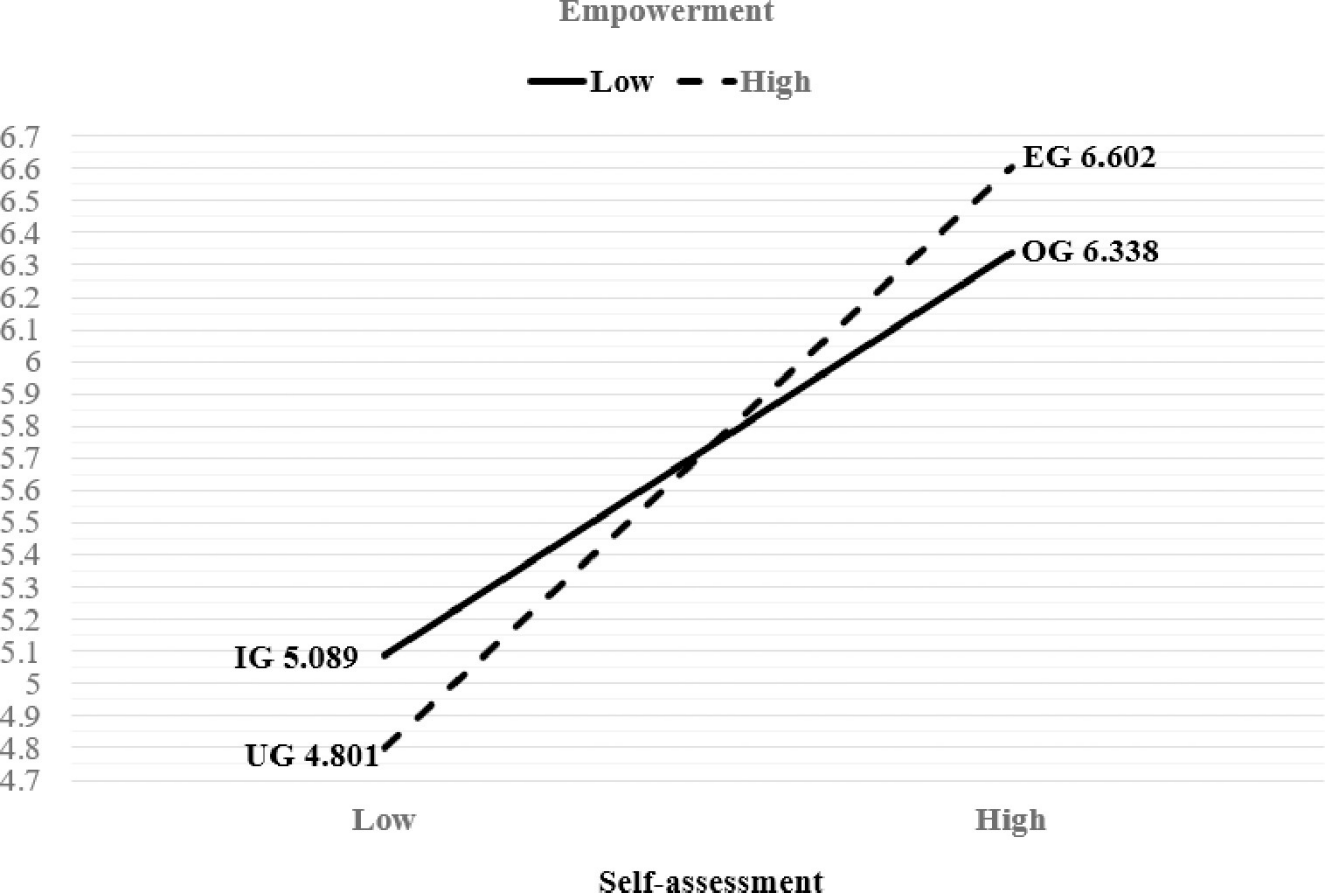
Effects of empowerment and self-assessment on information search behavior.

**Fig 3:**
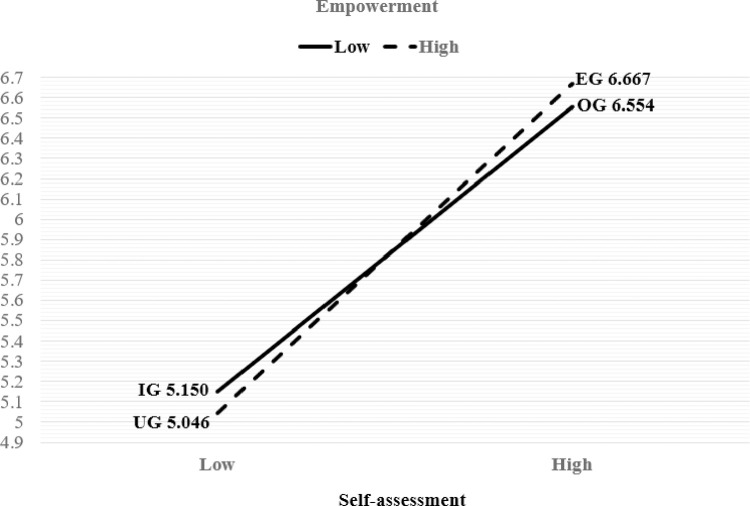
Effects of empowerment and self-assessment on consumer life satisfaction.

Table 4 and Figs 2 and 3 show the effects of empowerment and self-assessment on consumers’ information search behavior and consumer life satisfaction.

**Table 4:** Effects of empowerment and self-assessment on information search behavior and consumer life satisfaction.

	Information search behavior					Consumer life satisfaction				
	SS	df	F	p	$\eta_{p}^{2}$	SS	df	F	p	$\eta_{p}^{2}$
Male	.023	1	.016	.898	.000	.277	1	.170	.680	.000
Age	2.471	62	1.736	.001	.109	1.739	62	1.071	.336	.070
College and above	14.499	1	10.187	.001	.011	6.153	1	3.789	.052	.004
Mid income^a^	.479	1	.337	.562	.000	12.916	1	7.954	.005	.009
High income^b^	1.700	1	1.194	.275	.001	28.765	1	17.713	.000	.020
Empowerment (A)	.017	1	.012	.914	.000	.002	1	.002	.969	.000
Self-Assessment (B)	245.003	1	172.141	.000	.163	241.036	1	148.430	.000	.144
A×B	9.675	1	6.798	.009	.008	1.494	1	.920	.338	.001

Notes

^a^KRW 301–600

^b^KRW 601 and above.

Regarding information searching, the main effect of empowerment (SS = .017, F = .012, p = .914, Partialη^2^ = .000) was not statistically significant; therefore, Hypothesis 1 was not supported. However, age (SS = 2.471, F = 1.736, p = .001, Partialη^2^ = .109) and having a college education or higher (SS = 14.499, F = 10.187, p = .001, Partialη^2^ = .011) had a statistically significant positive influence on information search behavior, in addition to the main effect of self-assessment (SS = 245.003, F = 172.141, p = .000, Partialη^2^ = .163). The interaction effect of empowerment and self-assessment (SS = 9.675, F = 6.798, p = .009, Partialη^2^ = .008) had a statistically significant positive influence on information search behavior; therefore, Hypothesis 3 was supported. The groups with high self-assessment scores showed higher scores for information searching than the groups with low self-assessment scores. Among the low self-assessment groups, UG (M = 4.801, SE = .129), which had high empowerment scores, showed a lower information search score than IG (M = 5.089, SE = .172), which had low empowerment scores. Conversely, among the groups with high self-assessment scores, EG (M = 6.602, SE = .089), which had high empowerment scores, showed a higher information search score than OG (M = 6.338, SE = 0.99), which had low empowerment scores.

Regarding consumer life satisfaction, the mid-income (SS = 12.916, F = 7.954, p = .005, Partialη^2^ = .009) and high-income groups (SS = 28.765, F = 17.713, p = .000, Partialη^2^ = .020) had statistically significant positive influences on consumer life satisfaction. In addition, the main effect of self-assessment (SS = 241.036, F = 148.430, p = .000, Partialη^2^ = .144) had a statistically significant positive influence on consumer life satisfaction. However, the main effect of empowerment (SS = .002, F = .002, p = .969, Partialη^2^ = .000) and the interaction effect of empowerment and self-assessment (SS = 1.494, F = .920, p = .338, Partialη^2^ = .001) were not statistically significant. Therefore, Hypotheses 2 and 4 were not supported. Both EG (M = 6.667, SE = .095) and OG (M = 6.554, SE = .105), which had high self-assessment scores, showed higher scores for consumer life satisfaction than IG (M = 5.150, SE = .137) and UG (M = 5.046, SE = .184), which had low self-assessment scores.

## Discussion

In this study, the Dunning−Kruger effect on consumer empowerment was examined for 977 consumers aged over 20 years. Participants were classified according to their consumer empowerment and self-evaluation levels, using their responses from the 2018 Survey on Consumer Empowerment Index. Moreover, factors influencing the classification of consumers into these groups were investigated and the effects of consumer empowerment and self-assessment of empowerment on information search behavior and consumer life satisfaction were examined.

First, regarding the distribution of consumer groups according to their levels of empowerment and self-assessment, the largest group, comprising approximately half of the participants, was EG, which had both high empowerment and self-assessment; this was followed by OG, which had low empowerment but high self-assessment; IG, which had both low empowerment and self-assessment; and UG, which had high empowerment but low self-assessment. In this study, it appears that the reason the ratio of EG was relatively high is that the education level of the sample was comparatively high. All respondents in this study were high school graduates or higher, and 56.6% were college graduates. These results are consistent with previous research that showed that a higher education level precipitates higher consumer empowerment [48, 53, 54]. Although the Dunning−Kruger effect refers to the phenomenon in which people with low abilities overestimate their abilities, most studies applying the Dunning−Kruger effect also examine how people with high abilities underestimate their abilities [45]. The current study’s results indicate a Dunning−Kruger effect on consumer empowerment. This effect was seen in over one-third of the respondents who overestimated or underestimated their empowerment.

Second, regarding the factors that influenced the classification of participants into consumer groups, no factors were found to affect the likelihood of being classified as UG as compared to IG. However, younger consumers had a higher probability of being classified as OG compared to IG. In the case of EG, the influence of income and age was statistically significant. As OG and EG had high self-assessment scores, the results suggest that self-assessment is higher among younger consumers. This finding is consistent with the results of a previous study on genetically modified food by Hwang and Nam, who reported that younger people rated their knowledge of genetically modified food higher [48]. However, although previous studies [17, 48, 55] reported the influence of education on consumer knowledge or consumer empowerment, the influence of educational level was not verified in this study. In the results of the multinomial regression analysis, education level was divided into two groups: participants with a college degree or higher and those without a college degree. However, as shown in Table 1, only those who graduated from high school or higher were included in this study. Moreover, there was no difference between high school graduates and college graduates regarding consumer empowerment. The composition of this sample in terms of educational level can be attributed to the high level of education in Korea. According to the 2018 OECD Education at a Glance, 88% of Koreans aged 24–64 have graduated from high school [56].

Finally, this study showed that the main effect of consumer empowerment did not significantly affect consumers’ information searching or consumption satisfaction; however, the main effect of self-assessment did. In addition, there was an interaction effect of empowerment and self-assessment on information searching. Ultimately, self-assessment directly affected information search behavior and consumption satisfaction. However, the influence of empowerment on information searching and consumption satisfaction was not verified. Despite the significant interaction effect of empowerment with self-assessment on information search behavior, the $\eta_{p}^{2}$, which is a measure of effect size for use in ANOVA, was very low at .008. Therefore, an independent evaluation of the influence of empowerment and self-assessment on consumption behavior is worth considering. Brucks reported that subjective and objective knowledge, while correlated, cannot be substituted, and should be measured separately [57]. Similar to this study, other studies have shown that these two constructs have a weak to moderate relationship [27, 58–60].

The current study has some limitations. First, as the secondary analysis in this study was performed using the 2018 Survey on Consumer Empowerment Index, the ability to verify the influence of consumer empowerment and various consumer behaviors on self-assessment was limited. Therefore, it is necessary to verify aspects of consumption related to consumer empowerment and self-assessment in future studies. Second, as this study only included high school graduates, information on the empowerment of consumers with lower educational levels was not provided. Therefore, it is necessary to include analyses involving consumers with lower education in future studies.

## Supporting information

S1 Table Consumer knowledge. (DOCX)

S2 Table Consumer attitude and behavior. (DOCX)
