# Supplementary material for: Consumer empowerment and self-assessment of empowerment
Source: PLoS One. 2021 Nov 12;16(11):e0259971. doi: 10.1371/journal.pone.0259971 (PMC8589181; doi:10.1371/journal.pone.0259971)
Supplement: S1 Table — (DOCX) [file pone.0259971.s001.docx]

**S1 Table. Consumer Knowledge**

|  | T/F |
| --- | --- |
| 1. The user reviews posted on the Internet after using products or services are all objective consumer information. | False |
| 1. All products advertised in reliable media, such as TV and newspapers, have been confirmed to be safe. | False |
| 1. Online shopping or electronic financial transactions should not be conducted in public places and on computers shared with others. | True |
| 1. If all other conditions are the same, Shampoo A, which costs KRW 5,000 for 500g, is more economical than Shampoo B, which costs KRW 9,000 for 1,000g. | False |
| 1. If a youth under the age of 18 uses the Internet Contents Mall and pays the fee without the consent of their parents, the contract can be canceled in principle. | True |
| 1. Consumers who purchase products through e-commerce can cancel the contract within 7 days from the date of purchase. | True |
| 1. Quality comparison information increases consumer welfare by inducing price and quality competition among producers. | True |
| 1. If the quality guarantee period on the product is shorter than the period specified in the consumer dispute resolution standard, the period specified in the consumer dispute resolution standard for each denominator shall be followed. | True |
