# Supplementary material for: Consumer empowerment and self-assessment of empowerment
Source: PLoS One. 2021 Nov 12;16(11):e0259971. doi: 10.1371/journal.pone.0259971 (PMC8589181; doi:10.1371/journal.pone.0259971)
Supplement: S2 Table — (DOCX) [file pone.0259971.s002.docx]

**S2 Table. Consumer Attitude and Behavior**

| **Item** | **Factor 1** | **Factor 2** | **Factor 3** |
| --- | --- | --- | --- |
| **Consumer Attitude** |  |  |  |
| A1. When making an important purchase decision (e.g., TV, refrigerator, etc.), I think it is important to find enough consumer information before purchasing. | .207 | .070 | **.607** |
| A2. I think it is important to fully understand the meaning of the information (e.g., certification mark, country of origin) provided by the product. | **.458** | .130 | **.384** |
| A3. I think it is convenient to purchase goods and services using ICT (e.g., internet, mobile). | .369 | .026 | **.472** |
| A4. I don't think a high price guarantees the actual quality of product. | .121 | .105 | **.625** |
| A5. I think it is important to carefully compare the contents of contracts when purchasing products or services. | .132 | .199 | **.674** |
| A6. I think it is important to read the terms and conditions carefully when purchasing a service. | .185 | .228 | **.559** |
| A7. I think that comparing quality when purchasing products and services helps to make a reasonable purchase. | .136 | .409 | **.495** |
| A8. I think it is necessary to request an exchange or refund from the producer when there is a defect in the purchased product. | **-.048** | **.470** | **.533** |
| A9. I believe that as a user, I must fulfill the duty of care required for the safe use of the product. | .056 | .483 | **.561** |
| **Consumer Behavior** |  |  |  |
| B1. I. frequently use consumer information and related sites to make rational choices. | **.822** | -.014 | .076 |
| B2. When purchasing a product, I carefully check the displayed information labeled on the product. | **.596** | .245 | .237 |
| B3. When purchasing a product, I carefully check the displayed information labeled on the product. | **.788** | .157 | .116 |
| B4. When purchasing a product that will be used for a long time, I select it by considering maintenance costs in addition to the listed price in advance. | .400 | **.477** | .206 |
| B5. I refuse to recommend unnecessary purchases such as door-to-door sales, telephone ticket sales, and street sales. | .003 | **.588** | .129 |
| B6. When I buy a product, I compare and determine the price and quality of various products. | **.629** | .306 | -.038 |
| B7. I carefully read the terms and conditions of the transaction on the internet shopping mall. | .003 | **.588** | .129 |
| B8. When a consumer problem arises, I ask the producer for actions to be taken as a consumer, such as exchange, refund, and contract cancellation. | .202 | **.747** | .168 |
| B9. When using products, I obey the handling precautions of each product. | .181 | **.708** | .252 |
| Eigenvalue | 3.189 | 2.937 | 2.860 |
| Total variance explained, % | 17.716 | 16.319 | 15.891 |

**A1-A9: Consumer attitude, B1-B9: Consumer behavior**
